# Molecular Characterization of Ovarian Metastases from Colorectal Cancer Using Circulating Tumor DNA Analysis

**DOI:** 10.31662/jmaj.2026-0123

**Published:** 2026-05-22

**Authors:** Naoyuki Iwahashi, Tomoko Noguchi, Kazuko Sakai, Tamaki Yahata, Kumiko Nakata, Kaho Nishioka, Megumi Fujino, Shinichiro Takeda, Nobuhiko Suzuki, Kazuto Nishio, Kazuhiko Ino

**Affiliations:** 1 Department of Obstetrics and Gynecology, Wakayama Medical University, Wakayama, Japan; 2 Department of Genome Biology, Kindai University Faculty of Medicine, Osaka, Japan

**Keywords:** liquid biopsy, circulating tumor DNA, comprehensive genomic profiling, colorectal cancer, ovarian metastases

## Abstract

**Introduction:**

Ovarian metastases from colorectal cancer (CRC) are uncommon but are associated with poor prognosis and therapeutic challenges. These lesions may exhibit discordant responses to systemic therapy, suggesting site-specific biological heterogeneity. However, the molecular characteristics of CRC-associated ovarian metastases remain insufficiently defined. We aimed to characterize the circulating tumor deoxyribonucleic acid (ctDNA) landscape of ovarian metastatic CRC using a plasma-based deep sequencing approach.

**Methods:**

Plasma samples from patients with CRC and ovarian metastases were analyzed using cancer personalized profiling by deep sequencing. Pathogenic mutations and copy-number alterations were identified to delineate the genomic features of this metastatic subset.

**Results:**

Pathogenic genomic alterations were detected in the majority of patients. Canonical CRC driver mutations, including *TP53*, *KRAS*, and *APC*, were observed at frequencies comparable to those reported in large-scale genomic datasets such as The Cancer Genome Atlas. In addition, recurrent amplification of growth factor receptor genes, including *EGFR*, *MET*, and *ERBB2*, was identified. Detectable tumor-derived alterations in plasma indicate active ctDNA shedding from ovarian lesions, supporting the feasibility of noninvasive genomic profiling in this setting.

**Conclusions:**

CRC-associated ovarian metastases retain the core genomic architecture of colorectal tumorigenesis while demonstrating recurrent receptor tyrosine kinase amplifications that may reflect adaptive clonal evolution within the ovarian microenvironment. Plasma-based ctDNA profiling provides a feasible approach for molecular characterization of this rare metastatic phenotype and may contribute to improved understanding of site-specific tumor biology.

## Introduction

Ovarian metastases from colorectal cancer (CRC), commonly referred to as Krukenberg tumors, constitute a rare yet biologically distinctive manifestation of metastatic dissemination. Although their incidence is limited, ovarian involvement is consistently associated with aggressive clinical behavior and inferior survival relative to other metastatic sites ^(1), (2), (3)^. While retrospective series suggest that complete resection of isolated ovarian metastases may confer a survival benefit in selected patients ^(4), (5)^, therapeutic decision-making remains largely extrapolated from treatment paradigms developed for hepatic and pulmonary metastases, without specific consideration of ovarian tumor biology. Accumulating evidence indicates that metastatic CRC does not behave uniformly across anatomical compartments. Ovarian metastases, in particular, have been reported to demonstrate discordant therapeutic responses, with progression of ovarian lesions occurring despite disease control at extra-ovarian sites during systemic chemotherapy ^(3), (6)^. Such observations imply that the ovarian microenvironment may impose distinct selective pressures, influencing clonal expansion, drug exposure, and tumor evolutionary trajectories. This site-specific heterogeneity underscores the importance of molecular characterization rather than reliance solely on anatomical classification of metastases.

Circulating tumor deoxyribonucleic acid (ctDNA) analysis provides a minimally invasive platform for real-time assessment of tumor-derived genomic alterations and quantitative tumor burden. In CRC, postoperative ctDNA positivity has emerged as a robust marker of minimal residual disease and recurrence risk ^(7), (8), (9), (10)^. Beyond recurrence prediction, ctDNA profiling enables interrogation of clonal architecture and dynamic mutation patterns. However, the ctDNA landscape of rare metastatic phenotypes, including ovarian metastases, remains undefined. Whether ctDNA burden and mutation dynamics reflect the distinct biological behavior of ovarian metastatic CRC is unknown. A recent case report documented persistent postoperative ctDNA positivity preceding the diagnosis of bilateral ovarian metastases despite initially inconclusive imaging findings ^(11)^. Although suggestive, such single-case observations cannot delineate the molecular characteristics of ovarian metastatic disease at a cohort level.

In this study, we analyzed plasma ctDNA profiles from patients with CRC-associated ovarian metastases using a Cancer Personalized Profiling by deep sequencing (CAPP-seq)-based approach. CAPP-seq is an ultrasensitive next-generation sequencing methodology capable of detecting low-frequency tumor-derived variants with high quantitative precision ^(12), (13)^. Application of CAPP-seq to ovarian metastatic CRC offers an opportunity to systematically characterize ctDNA detectability, mutation burden, and longitudinal clonal dynamics within this uncommon but clinically consequential metastatic subset. This study aimed to characterize the ctDNA landscape of colorectal cancer-associated ovarian metastases using a plasma-based sequencing approach.

## Materials and Methods

### Participants

We retrospectively enrolled 11 women diagnosed with ovarian metastases from CRC who underwent surgical resection between July 2017 and August 2020 at Wakayama Medical University Hospital, Japan. Blood specimens were obtained prior to surgery. Approval was granted by the ethical committees of Wakayama Medical University (authorization number: 2025) and Kindai University Faculty of Medicine (authorization number: 29-066). Written informed consent was obtained from each participant, and the study adhered to the principles of the 2008 revision of the Declaration of Helsinki.

### Circulating tumor DNA extraction

For cell-free DNA (cfDNA) isolation, peripheral blood (8.5 mL) was drawn into cfDNA blood collection tubes (Roche Diagnostics, Indianapolis, IN, USA). From each sample, 4 mL of plasma was processed with the AVENIO cfDNA isolation kit (Roche Diagnostics) according to the manufacturer’s protocol, and ctDNA was eluted in 65 μL of elution buffer. Concentration and quality of the extracted ctDNA were verified using the PicoGreen dsDNA assay kit (Life Technologies, Carlsbad, CA, USA) according to the manufacturer’s protocol. DNA mass measured in nanograms was converted to genome equivalents, assuming that one haploid human genome corresponds to 3.3 pg of DNA (approximately 303 genome equivalents per ng), as recommended in the manufacturer’s documentation and commonly applied in clinical cfDNA studies. Samples were stored at −80°C until use.

### Circulating tumor DNA sequencing

CAPP-seq was performed on 10-50 ng of ctDNA using the AVENIO ctDNA Surveillance Kit, which targets 197 genes (Roche Diagnostics), as previously described ^(14), (15)^. Purified libraries were then pooled and sequenced on an Illumina NextSeq 500 system with a 300-cycle high-output kit (Illumina, San Diego, CA, USA). Variant calling utilized the AVENIO ctDNA Analysis Software (Roche Diagnostics), which integrates the CAPP-seq pipeline ^(12)^ and digital error suppression ^(13)^. Variants found at ≥1% frequency in the Exome Aggregation Consortium were eliminated, and only non-synonymous single-nucleotide variants, insertions/deletions, copy-number alterations, and gene fusions related to the 197 genes were reported. Blood tumor mutational burden (bTMB) was determined as the number of non-synonymous mutations per megabase.

### Immunohistochemistry

Paraffin-embedded tissue blocks were cut into 3-μm-thick sections, after which the sections were deparaffinized and rehydrated. Epitopes were then retrieved by heat-induced antigen retrieval―boiling the sections in a pressure cooker in citrate buffer (10 mM sodium citrate, 0.05% Tween 20, pH 6.0) for 20 min. The DO-7 mouse monoclonal anti-p53 antibody that was used for p53 immunohistochemical evaluation of ovarian carcinoma ^(16)^ served as the primary antibody (1:100). Sections were then incubated with a horseradish peroxidase-labeled goat anti-mouse secondary antibody (Histofine; Nichirei Biosciences, Tokyo, Japan). Signals were detected using a diaminobenzidine substrate kit (Nichirei Biosciences). All slides were independently evaluated by experienced pathologists who were blinded to the ctDNA results.

### Statistical analysis

Statistical analyses were conducted using Prism software (GraphPad Software, version 10.6.1; San Diego, CA, USA). The correlation between cfDNA concentration and bTMB was evaluated using Spearman’s rank correlation coefficient, with p-values <0.05 considered statistically significant.

## Results

### Pretreatment cfDNA levels and bTMB

**Figure 1.**
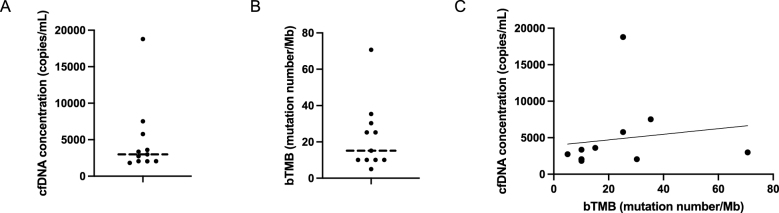
Distribution of plasma cfDNA concentration and plasma bTMB in ovarian metastases from colorectal cancer. A) Plasma cfDNA concentration; B) plasma bTMB concentration; C) association between plasma cfDNA concentration and plasma bTMB concentration. cfDNA concentration is expressed as genome equivalents per milliliter, calculated from PicoGreen-measured DNA mass according to the manufacturer’s protocol. bTMB is expressed as the number of somatic mutations per megabase (mutations/Mb), derived from ctDNA sequencing data obtained by CAPP-seq analysis. Statistical comparisons were performed using Spearman’s rank correlation test for comparison (C). bTMB: blood tumor mutational burden; CAPP-seq: cancer personalized profiling by deep sequencing; cfDNA: cell-free deoxyribonucleic acid.

We retrospectively studied 11 women with colorectal cancer-associated ovarian metastases (median age, 50 years [range, 38-72]; median body mass index, 21.9 kg/m^2^ [range, 20.0-29.5]). We evaluated the cfDNA concentration and bTMB in pretreatment plasma samples (Figure 1). The median cfDNA level was 2,999.9 copies/mL (range, 1,822.1-18,789.9), and the median bTMB was 15.2 mutations/Mb (range, 5.0-70.7). Spearman’s rank correlation analysis showed a moderate but non-significant association between cfDNA concentration and bTMB (ρ = 0.52, p = 0.10). In a sensitivity analysis excluding case OVO-104, which had an outlying cfDNA value, the association strengthened and reached statistical significance (ρ = 0.67, p = 0.034).

### Comprehensive genomic profiling of CRC-associated ovarian metastases using CAPP-seq

**Figure 2.**
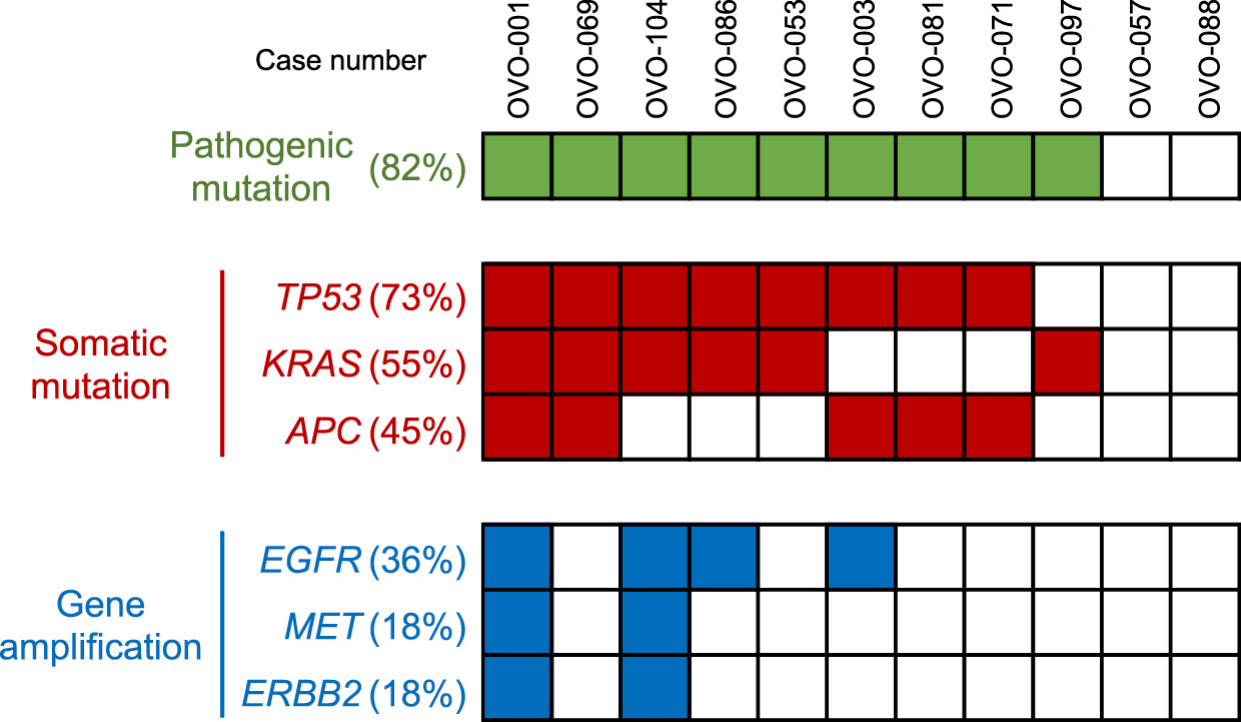
Comprehensive profiling of genetic alterations in 11 patients with ovarian metastases from colorectal cancer using liquid biopsy-based CAPP-seq. The figure summarizes plasma-based genomic profiling, the presence or absence of pathogenic mutations, major somatic mutations, and copy number alterations detected by ctDNA analysis. Each column represents an individual patient, and genetic alterations are displayed according to gene and alteration type. ctDNA: circulating tumor deoxyribonucleic acid; CAPP-seq, cancer personalized profiling by deep sequencing.

Next, we performed comprehensive genomic profiling of circulating tumor DNA in pretreatment plasma samples from patients with CRC-associated ovarian metastases using a 197-gene CAPP-seq panel. The targeted 197-gene CAPP-seq panel used in this study did not include genes associated with clonal hematopoiesis of indeterminate potential (CHIP). A complete array of identified alterations, covering pathogenic mutations and gene amplification, is shown in Figure 2. Overall, 9 of 11 patients (82%) carried ≥1 non-synonymous somatic pathogenic mutation, most frequently in *TP53* [8/11 (73%)], *KRAS* [6/11 (55%)], and *APC* [5/11 (45%)], which was concordant with The Cancer Genome Atlas (TCGA) database ^(17)^. We also detected somatic copy-number amplification in three genes, *EGFR*, *MET*, and *ERBB2*, in plasma ctDNA using CAPP-seq. *EGFR* copy-number amplifications were observed in four patients (36%), *MET* copy-number amplifications were observed in two patients (18%), and *ERBB2* copy-number amplifications were observed in two patients (18%).

### Concordance of TP53 mutations between ctDNA and tissue immunohistochemistry

Given that p53 immunohistochemistry (IHC) has been used as a surrogate for *TP53* mutational status in CRC ^(18), (19)^, we evaluated the concordance between p53 IHC expression patterns and *TP53* gene alterations in this cohort (Table 1). The overall concordance between p53 IHC patterns and *TP53* mutation status was 90.9% (10/11) (Table 2). Although the sample size was limited, when ctDNA-detected *TP53* mutation was used as the reference, p53 immunohistochemistry achieved a sensitivity of 100% (95% confidence interval [CI], 63.1%-100%) and a specificity of 66.7% (95% CI, 9.4%-99.2%). These findings suggest that ctDNA-based analysis may provide a reliable and clinically feasible approach for detecting *TP53* mutations and could serve as a practical tool for molecular characterization, particularly when tissue access is limited.

**Table 1.**
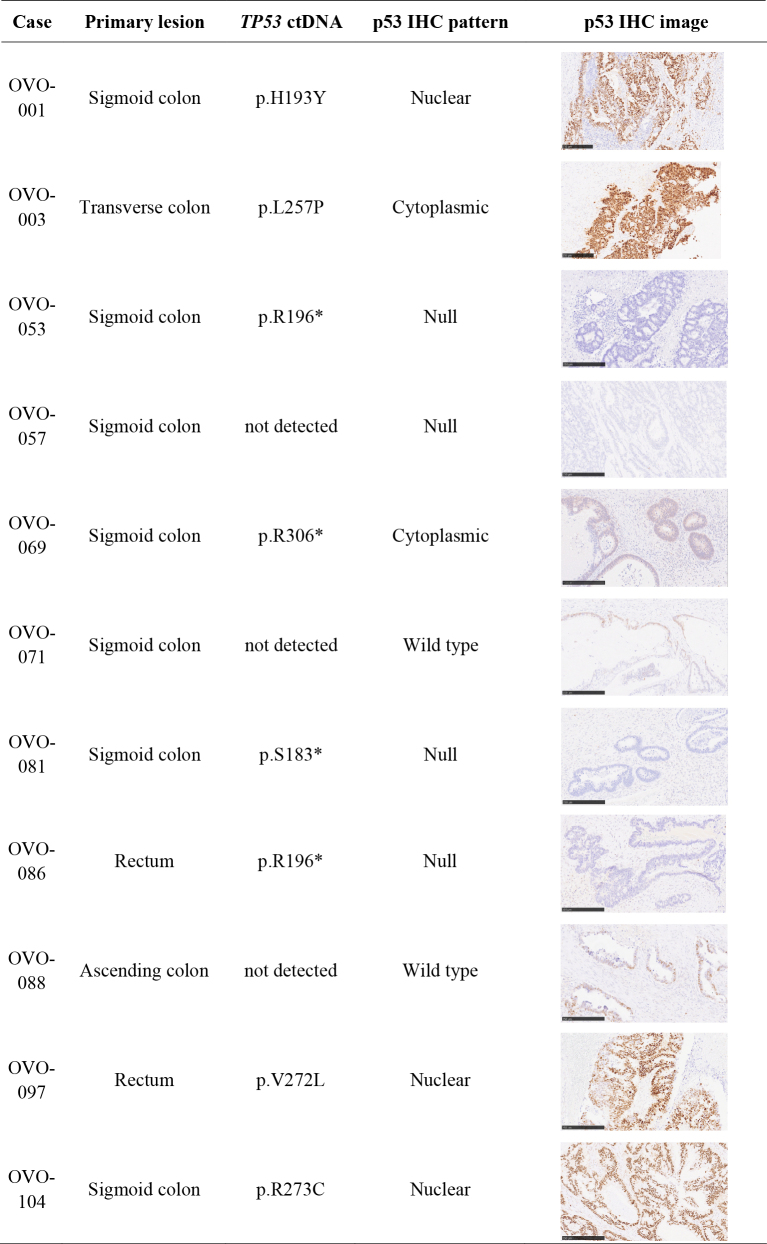
Concordance between *TP53* mutation status in ctDNA and p53 immunohistochemical expression patterns.

**Table 2.** Sensitivity and specificity between *TP53* mutation status in ctDNA and p53 immunohistochemical expression patterns.

	p53 IHC abnormal	p53 IHC wild type	total
ctDNA *TP53* mutation (+)	8	0	8
ctDNA *TP53* mutation (-)	1	2	3
total	9	2	11

## Discussion

This study provides a molecular characterization of CRC-associated ovarian metastases using CAPP-seq-based circulating tumor DNA (ctDNA) analysis. Ovarian metastases from CRC are uncommon but are consistently associated with poor prognosis and therapeutic challenges ^(2), (3)^ Although selected patients may benefit from metastatectomy ^(4), (5)^, ovarian lesions frequently progress despite apparent systemic disease control ^(1)^ This discordant behavior has often been attributed to pharmacokinetic factors; however, accumulating evidence suggests that organ-specific microenvironments influence clonal selection and metastatic evolution through “seed and soil” interactions ^(20), (21)^. The ovarian stroma, characterized by hormonally responsive tissue architecture and dynamic vascular remodeling, may impose ecological constraints distinct from hepatic or pulmonary metastatic niches. In this context, sensitive molecular interrogation is essential to elucidate subclonal complexity and adaptive evolutionary processes.

CAPP-seq is an ultrasensitive, tumor-informed deep sequencing platform originally developed to detect low-frequency circulating tumor DNA alterations across solid tumors ^(12)^. Subsequent refinements have improved background error suppression and broadened clinical applicability ^(13)^. We previously demonstrated the feasibility of CAPP-seq-based ctDNA profiling in gynecologic malignancies, including ovarian cancer ^(14), (15)^, and subsequently extended this approach to CRC-associated ovarian metastases. In parallel, plasma-based deep sequencing strategies have been validated in CRC, particularly for minimal residual disease detection and recurrence monitoring ^(7), (8), (9), (10), (22)^. Collectively, these studies establish the analytical validity of high-sensitivity ctDNA profiling in CRC and provide the foundation for its application to rare metastatic phenotypes. In the present cohort, canonical CRC driver mutations―including *TP53*, *KRAS*, and *APC*―were detected at frequencies comparable to those reported in the TCGA colon and rectal cancer dataset ^(22)^. In TCGA, *APC* alterations occur in approximately 75% of cases, *TP53* mutations in 60%, and *KRAS* mutations in 40%, defining the core genomic architecture of colorectal tumorigenesis. The concordance of our findings with these large-scale genomic data supports lineage continuity between primary colorectal tumors and ovarian metastases and argues against the emergence of a distinct ovarian-specific molecular subtype. Instead, ovarian metastases appear to retain the canonical genomic backbone of CRC while potentially acquiring additional alterations during metastatic progression.

Our data further demonstrate that plasma-based genomic profiling is biologically informative in this uncommon metastatic subset. Detectable tumor-derived alterations in most patients indicate active ctDNA shedding from ovarian lesions, challenging the assumption that sanctuary-like compartments necessarily contribute minimally to circulating tumor DNA. Consistent with prior evidence that ctDNA reflects metastatic tumor burden in advanced malignancies ^(23)^, ovarian metastases appear capable of releasing measurable tumor DNA into the systemic circulation, enabling noninvasive genomic interrogation when tissue access is limited. Beyond detectability, ctDNA profiling provides insight into clonal architecture and metastatic evolution. The coexistence of canonical colorectal driver mutations with recurrent amplification of growth factor receptor genes―including *EGFR*, *MET*, and *ERBB2*―is consistent with a branched evolutionary model of metastatic dissemination ^(24)^. Amplification of receptor tyrosine kinases has been linked to enhanced proliferative signaling and resistance mechanisms in metastatic CRC ^(25), (26)^. Notably, *ERBB2* (HER2) amplification has become clinically actionable with the development of HER2-targeted therapeutic strategies in RAS wild-type metastatic CRC, including dual HER2 blockade and antibody-drug conjugates such as trastuzumab deruxtecan ^(27), (28)^, underscoring the translational relevance of comprehensive genomic profiling. Under selective pressures imposed by systemic therapy and the ovarian microenvironment, subclonal populations harboring amplified signaling pathways may be preferentially expanded, facilitating tumor persistence. Longitudinal ctDNA monitoring will therefore be important to determine whether these alterations represent stable genomic features or dynamic adaptive events emerging during treatment.

Several limitations should be acknowledged. The rarity of ovarian metastatic CRC limited the cohort size and statistical power, restricting definitive conclusions regarding alteration enrichment and generalizability. Matched tissue sequencing was not uniformly available, preventing direct evaluation of concordance and phylogenetic relationships between primary and metastatic lesions using identical gene panels. Although the gene panel did not primarily target common CHIP-associated genes, the absence of leukocyte controls limited the complete exclusion of rare non-tumor variants. Nevertheless, this work represents an initial effort to define the ctDNA landscape of ovarian metastatic CRC using a clinically applicable deep sequencing platform.

In conclusion, CRC-associated ovarian metastases retain canonical colorectal driver mutations while demonstrating recurrent receptor tyrosine kinase amplifications that may reflect adaptive clonal evolution within the ovarian microenvironment. CAPP-seq-based ctDNA profiling provides a feasible and informative strategy for molecular characterization of this rare metastatic phenotype and may contribute to improved understanding of site-specific tumor biology and therapeutic decision-making.

## Article Information

### Acknowledgments

This article is based on the study, which received the Medical Research Encouragement Prize of the Japan Medical Association in 2025.

### Author Contributions

Conceptualization: Naoyuki Iwahashi, Kazuto Nishio, and Kazuhiko Ino. Methodology: Tomoko Noguchi and Kazuko Sakai. Formal analysis: Naoyuki Iwahashi. Investigation: Naoyuki Iwahashi and Tomoko Noguchi. Resources: Tamaki Yahata, Kumiko Nakata, Kaho Nishioka, Megumi Fujino, Shinichiro Takeda, and Nobuhiko Suzuki. Data curation: Naoyuki Iwahashi. Writing―original draft preparation: Naoyuki Iwahashi. Writing―review and editing: Kazuko Sakai, Kazuto Nishio, and Kazuhiko Ino. Visualization: Naoyuki Iwahashi and Tomoko Noguchi. Supervision: Kazuto Nishio and Kazuhiko Ino. Project administration: Naoyuki Iwahashi. Funding acquisition: Naoyuki Iwahashi and Kazuhiko Ino. All authors have read and agreed to the published version of the manuscript.

### Conflicts of Interest

None

### IRB Approval Code and Name of the Institution

Approval was granted by the ethical committees of Wakayama Medical University (authorization number: 2026) and Kindai University Faculty of Medicine (authorization number: 29-066).
